# Neighbourhood Deprivation and Outcomes of Stop Smoking Support – An Observational Study

**DOI:** 10.1371/journal.pone.0148194

**Published:** 2016-01-29

**Authors:** Leonie S. Brose, Andy McEwen

**Affiliations:** 1 National Addiction Centre & UK Centre for Tobacco and Alcohol Studies, Institute of Psychiatry, Psychology and Neuroscience, King’s College London, London, United Kingdom; 2 National Centre for Smoking Cessation and Training & Cancer Research UK Health Behaviour Research Centre, University College London, London, United Kingdom; BRAC, BANGLADESH

## Abstract

**Background:**

Rates of smoking and smoking cessation vary with socio-economic status. The objectives were to assess the association between neighbourhood deprivation, completion of treatment to support quit attempts and success of quit attempts—while taking into account other predictors of outcome.

**Methods:**

555,744 quit attempts supported by English Stop Smoking Services in 2009–2012 were linked to the Index of Multiple Deprivation (IMD) 2010 ranks for the clients’ neighbourhood and split into deciles relative to the national IMD. Logistic regressions tested the association between neighbourhood deprivation and completion (4-week follow-up) of treatment and biochemically validated success (expired-air carbon monoxide <10ppm) while adjusting for demographics and intervention characteristics. Sensitivity analyses assessed subsamples: first supported attempts (n = 364,397), those with recorded cigarette dependence (n = 98,659) and completed treatment (n = 416,436).

**Results:**

Higher neighbourhood deprivation was associated with reduced completion (OR_adj_ = 0.949, 95% CI: 0.947 to 0.951) and success (OR_adj_ = 0.957, 95% CI: 0.955 to 0.959). Results of sensitivity analyses were consistent with those of the main analysis.

**Conclusions:**

Neighbourhood deprivation was associated with small but consistent reductions in completion and success of evidence-based interventions. These associations were not explained by intervention characteristics, demographics or dependence and reduced completion did not fully account for reduced success.

## Introduction

Smoking is a major determinant of health inequalities [1] and increased efforts tackling smoking are required to reverse the trend of increasing health inequalities in Europe [2].

In England, those living in the most deprived areas are about 2.5 times more likely to smoke than those living in the least deprived areas [3], which contributes to a gap in life expectancy of up to 17 years [4]. A number of studies from different countries using a range of indicators have found this effect of neighbourhood deprivation to be independent of individual deprivation [5–8]. People living in the least deprived areas are far more likely to be former smokers, i.e. have successfully quit smoking [3], an association also found across Europe [9]. Residents in these areas are less likely to be ex-smokers not because they are less likely to make attempts to quit smoking but because these attempts are less likely to succeed [10–12].

Rates of success in quitting can be enhanced considerably with adequate medication [13] and behavioural support, particularly in combination [14], as provided by Stop Smoking Services in England for example [15]. The services, which as part of the National Health Service (NHS) are free at the point of access, have been shown to be an extremely cost-effective life-saving intervention [16].

In contrast to the inverse-care law hypothesis that populations most in need of healthcare are least likely to receive it [17], evidence indicates that Stop Smoking Services have been able to support disadvantaged smokers. A first evaluation of a fifth of the services in 2001 described good reach in more deprived areas [18], with reach defined as the number of service users setting a quit date as a proportion of the adult smoking population by level of deprivation. Good reach was also indicated in more recent evaluations using exemption from prescription charges as a proxy measure for deprivation [16]. Hence, despite most smokers attempting to quit doing so without accessing the services, it was concluded that these services made a modest contribution to reducing inequalities in smoking prevalence [19, 20]. However, although services in more deprived areas had better reach than those in less deprived areas, they were also more likely to lose contact with clients [21]. Data from two areas in England collected in 2001–2003 also showed that ‘rather than quitting smoking, disadvantaged smokers quit treatment’[22], an association that may also be apparent in other countries [23]. It is unclear if the association between deprivation and reduced completion and success of smoking cessation treatment in England has improved since these early findings and whether this association is found in a wider range of regions.

This study aimed to assess the association between neighbourhood deprivation and a) 4-week completion of smoking cessation treatment and b) quit success while adjusting for other predictors and improving on previous studies by using a more recent, larger data set from across the country.

## Methods

### Sample

The initial sample consisted of 646,590 quit attempts supported by English Stop Smoking Services completed between April 2009 and April 2012; 33,997 still in progress were excluded along with 11,574 which were missing information on completion status and 24,452 with missing or non-existing postcodes. Attempts to quit were also excluded where treatment was delivered over the phone (n = 10,956), as outcome can rarely be biochemically validated (carbon monoxide in exhaled breath cannot be measured over the phone), or delivered in prison (n = 7,622), where postcode information was not meaningful. Finally, 2,245 quit attempts missing information on key demographic or intervention characteristics were excluded, resulting in N = 555,744 (85.95% of the initial sample) retained for analysis (S1 Dataset). Ethical approval for secondary analysis of these anonymised data routinely collected in clinical practice was granted by King’s College London Psychiatry, Nursing and Midwifery Research Ethics Subcommittee.

### Measures

#### Demographic and intervention characteristics

Demographic and intervention characteristics were obtained from data routinely collected in clinical practice using QuitManager (North51, Nottingham, UK), an online database for recording information on client demographics, intervention characteristics and outcomes.

Demographics, other than deprivation measures covered below, were gender and age at quit date. Tobacco dependence as measured by the Heaviness-of-Smoking Index, with higher scores (range 0–6) indicating higher levels of dependence, is an optional clinical measure, was recorded for only about 18% of the sample and was analysed separately.

Intervention characteristics included medication (no medication, single nicotine replacement therapy [NRT], combination NRT, bupropion, varenicline), intervention type (scheduled one-to-one appointments, more flexible drop-in, group, ‘other’ such as couple/family sessions), intervention setting (specialist clinics, primary care, pharmacy and ‘other’), and whether a quit attempt was either a client’s first, or repeated attempt, supported by the service.

#### Deprivation

Neighbourhood deprivation: Neighbourhoods were defined using Lower Layer Super Output Areas (LSOA), small areas of relatively even size containing approximately 1,500 people. For each LSOA, an Index of Multiple Deprivation (IMD) is available. The IMD is made up of seven domain indices related to income, employment, health and disability, education and training, barriers to housing and services, living environment, and crime, which aim to reflect the broad range of deprivation that people can experience. The domains are transformed and combined using appropriate weights to derive the overall IMD [24]. Using clients’ home postcodes, each client was linked to their LSOA, which in turn was linked to their overall IMD and IMD domain ranks. Data linkage was completed using Matlab. Clients were grouped into ten deprivation categories based on the ranking of the IMD rank of their neighbourhood in relation to deciles of all LSOA ranks in England.

Individual deprivation: Occupational status as measured by the National Statistics Socio-Economic Classification (NS-SEC) was dichotomized into employed (professional/managerial, intermediate, routine/manual, full-time student, retired), and not employed (never worked/long-term unemployed, home carer, sick/disabled and unable to work, other). As a rough proxy measure, exemption from paying NHS prescription charges was also used. Patients on benefits are exempt from paying NHS prescription charges, but the utility of this measure as a deprivation measure is limited as exemption criteria also include age under 16 or over 60 years, pregnancy and chronic conditions [25, 26]. Other potential measures of socio-economic status, for example education or income, were not available.

#### Outcomes

The two primary outcome measures were completion and quit success. Completion means that the client had completed the mandatory 4-week follow-up. It is standard to define those lost to follow-up as having resumed smoking, thus completion directly impacts success rates [27]. Validated quit success as defined by the Russell Standard (Clinical) is recorded if at follow-up the smoker reports continuous abstinence between weeks 2 and 4 and records an expired-air carbon monoxide reading of <10 parts per million [27]. Self-reported success, whether or not validated biochemically, was included as additional outcome in descriptive analyses.

### Analysis

Analyses were conducted using SPSS 22. Demographic and intervention characteristics and outcome measures across neighbourhood deprivation deciles were described using proportions and means with standard deviations. Linearity of trends across deciles was tested using one-way ANOVAs.

Bivariate and multivariable logistic regressions with a) completion and b) quit success as outcomes and neighbourhood IMD as a predictor. Multivariable regressions adjusted for intervention characteristics and demographics. An initial model included age and gender as demographics, a second model included occupational grade and exemption from prescription charges. For the second model, blockwise entry was used and contribution of blocks tested using 2Log-Likelihood and chi-square statistics. Multicollinearity was assessed using tolerance and VIF values from equivalent linear regressions [28].

Additional logistic regressions were conducted as sensitivity analyses based on the second model for a) completed treatment, b) the subsample with information on cigarette dependence, both with and without dependence as an additional predictor and c) clients’ first supported attempts only. The latter was conducted because in some cases, more than one quit attempt may have been recorded for the same individual within the dataset. The subsample undertaking their first attempt excludes anyone recorded twice and anyone whose previous supported attempt was undertaken outside of the recorded data.

## Results

### Sample characteristics

**Fig 1:**
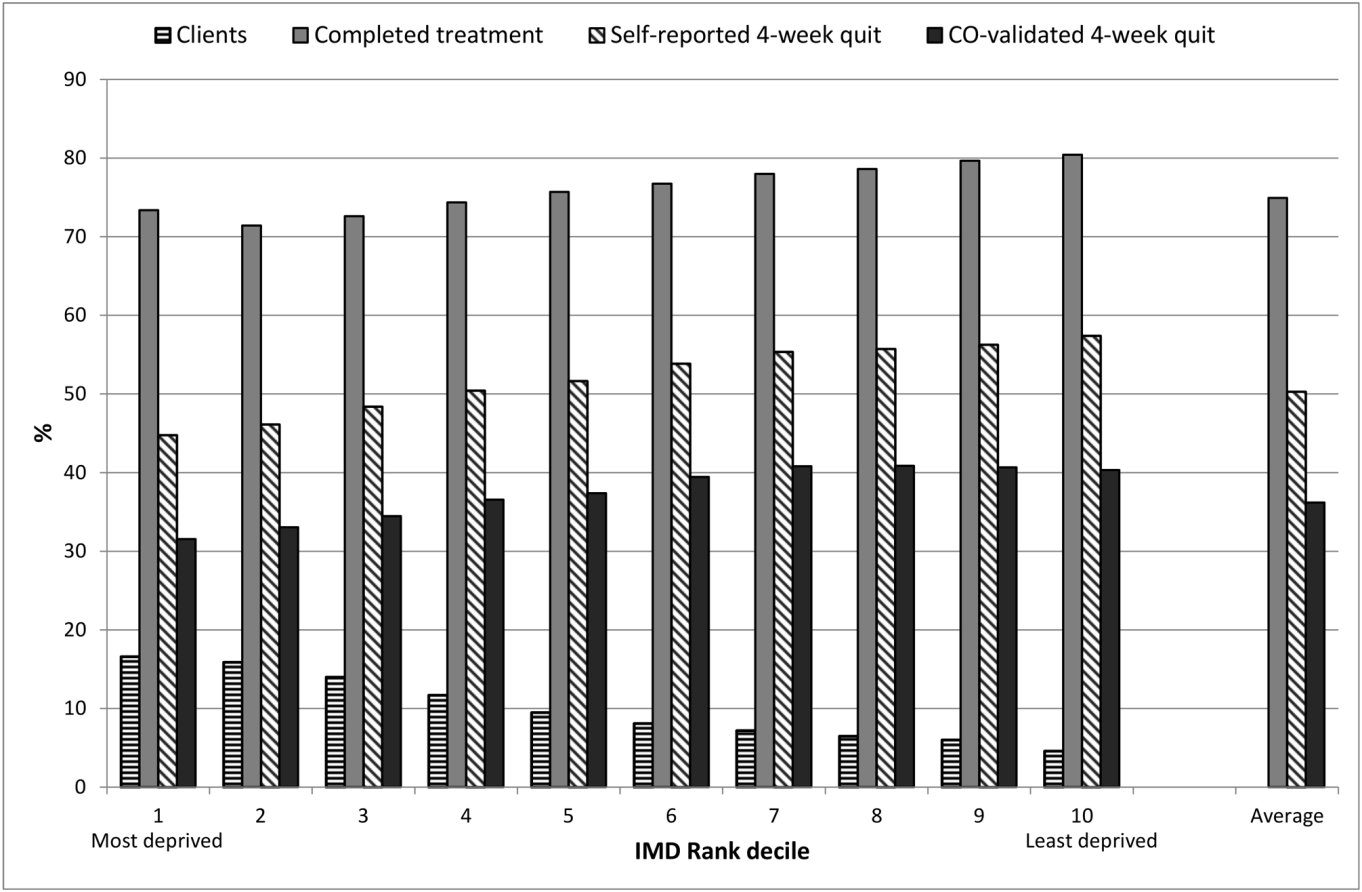
Clients and outcomes by deciles of deprivation.

The proportion of clients increased with increased neighbourhood deprivation (Fig 1, Table 1). Demographic and intervention characteristics were distributed unevenly across levels of neighbourhood deprivation. Notable differences included that, while overall the same proportion used combination NRT as varenicline (the two most effective medication options), combination NRT use increased with neighbourhood deprivation (F(9,555734) = 284.99, p<0.001, linear trend p<0.001); varenicline use decreased (Table 1, F(9,555734) = 454.70, p<0.001, linear trend p<0.001). Clients from more deprived neighbourhoods were more likely to be attending for a repeated attempt supported by the service than clients from less deprived neighbourhoods (Table 1, F(9,555734) = 514.25, p<0.001, linear trend p<0.001). In the subsample with dependence recorded, dependence increased in line with neighbourhood deprivation (F(9,98649) = 129.57, p<0.001, linear trend p<0.001); however, recording was also not consistent across groups (Table 1).

**Table 1:** Client demographic and intervention characteristics by level of deprivation.

IMD decile ^a^	N	Age	Men	Em-ployed ^b^	Pays for prescript-tion	1^st^ supported attempt	Heaviness of Smoking Index		Medication ^c^				Group support	Specialist clinic
		M (SD)	%	%	%	%	M (SD)	Recorded, %	None, %	Single NRT, %	Combi NRT, %	Vareni-cline, %	%	%
**1**	92174	41.25 (14.95)	46.2	48.2	21.4	59.6	3.55 (1.43)	20.5	8.3	30.8	39.1	21.1	4.4	41.4
**2**	88307	41.83 (14.86)	45.3	52.0	27.1	62.6	3.45 (1.46)	20.3	11.3	30.2	35.0	22.7	3.8	33.5
**3**	77704	42.18 (14.82)	46.1	55.8	31.2	63.8	3.38 (1.44)	17.8	11.3	29.6	33.3	24.9	4.4	32.5
**4**	64919	42.50 (14.98)	45.8	61.8	34.4	64.8	3.29 (1.46)	16.7	9.4	29.6	33.2	26.7	4.7	33.6
**5**	52901	42.75 (14.96)	46.5	64.9	36.4	66.6	3.28 (1.43)	16.1	9.5	28.8	32.9	27.8	4.7	34.2
**6**	45262	43.13 (15.08)	46.8	68.6	39.0	67.8	3.21 (1.46)	16.6	8.5	29.0	31.6	29.9	5.2	33.6
**7**	39951	43.69 (15.12)	47.7	70.5	40.8	69.9	3.19 (1.43)	16.4	8.5	29.0	30.7	30.7	5.0	32.1
**8**	35847	43.58 (15.04)	48.2	73.5	42.9	70.5	3.13 (1.44)	15.9	8.0	29.4	29.6	31.7	5.6	31.6
**9**	33360	43.87 (15.09)	49.2	74.8	45.1	73.0	3.10 (1.45)	16.7	8.9	29.1	28.6	32.1	4.3	29.9
**10**	25319	43.45 (15.23)	50.5	77.2	46.4	75.1	2.98 (1.43)	13.1	10.3	29.9	28.1	30.6	3.3	28.8

^a^ IMD deciles: 1 –most deprived, 10 –least deprived;

^b^ Including students and retired clients.

^c^ Bupropion not shown, rates between 0.7% and 1.3%.

### Association between neighbourhood deprivation and outcomes

Completion rates decreased as neighbourhood deprivation increased and self-reported and biochemically validated success rates were slightly reduced by an increase in neighbourhood deprivation (Table 1, Fig 1, unadjusted results for change in deprivation: Completion OR = 0.949, 95% CI = 0.947 to 0.951; success OR = 0.950, 95% CI: 0.948 to 0.952).

When adjusting for other predictors, neighbourhood deprivation remained associated with a small reduction in completion and success of treatment (Table 2).

**Table 2:** Adjusted association between client demographic and intervention characteristics and outcomes.

	Completion						Quit success (CO-validated)					
	Model 1			Model 2			Model 1			Model 2		
	OR	95% CI		OR	95% CI		OR	95% CI		OR	95% CI	
Neighbourhood deprivation (per IMD decile)	0.949	0.947	0.951	0.955	0.953	0.958	0.957	0.955	0.959	0.968	0.966	0.970
Men compared with women	0.973	0.961	0.985	0.959	0.947	0.971	1.040	1.028	1.052	1.016	1.005	1.028
Age (per year increase)	1.022	1.021	1.022	1.022	1.021	1.022	1.018	1.018	1.019	1.018	1.018	1.019
Later compared with first supported attempt	1.090	1.075	1.105	1.098	1.083	1.113	0.947	0.936	0.959	0.962	0.950	0.973
Medication, reference none												
Single NRT	1.119	1.095	1.143	1.119	1.095	1.144	1.487	1.453	1.523	1.473	1.439	1.508
Bupropion	1.539	1.438	1.648	1.502	1.403	1.609	1.906	1.793	2.026	1.808	1.700	1.922
Combination NRT	1.613	1.579	1.649	1.617	1.581	1.653	2.295	2.242	2.348	2.283	2.230	2.337
Varenicline	1.719	1.680	1.758	1.676	1.637	1.715	2.918	2.851	2.988	2.764	2.699	2.830
Intervention type, reference one-to-one												
Group	1.648	1.585	1.713	1.635	1.573	1.700	1.642	1.597	1.688	1.618	1.573	1.663
Drop-in	1.142	1.116	1.168	1.135	1.110	1.162	0.917	0.899	0.935	0.908	0.890	0.925
Other	1.265	1.186	1.349	1.280	1.201	1.365	1.182	1.119	1.249	1.207	1.142	1.275
Intervention setting, reference specialist service												
Primary care	0.656	0.645	0.668	0.659	0.647	0.670	0.812	0.799	0.824	0.817	0.804	0.830
Pharmacy	0.597	0.585	0.609	0.601	0.589	0.614	1.055	1.036	1.074	1.062	1.044	1.082
Other	1.136	1.095	1.179	1.129	1.088	1.171	0.930	0.901	0.960	0.921	0.892	0.951
Occupational status, reference employed												
Not employed				0.873	0.860	0.887				0.793	0.781	0.804
Unable to code				0.844	0.828	0.860				0.806	0.792	0.821
Prescription charge, reference pays												
Exempt				0.924	0.910	0.938				0.840	0.829	0.851
Unknown				0.969	0.948	0.991				0.811	0.793	0.828

Although the inclusion of additional individual indicators of deprivation reduced the size of the association, neighbourhood deprivation remained associated with both outcomes. Additional individual indicators were also significantly associated with outcomes: not being in employment and being exempt from prescription charges both significantly reduced completion and quit success while adjusting for all other predictors (Table 2). Occupational status and exemption status made a significant contribution to the models for both outcome measures as indicated by 2Log-Likelihood and chi-square statistics (all p<0.001).

No multicollinearity issues were detected for either model. All predictors had tolerance values of at least 0.95 and VIF values below 1.06, thus well clear of critical values of VIF <0.1 or VIF>10 [28]. Condition indices (all <30) and variance proportions did not indicate multicollinearity either.

#### Sensitivity analyses

Although differences in completion rates to a large extent explained differences in success rates across neighbourhood deprivation deciles in a simple linear regression (R^2^ = 0.83), the association between success and neighbourhood deprivation remained detectable in the analysis including only clients who had completed treatment (n = 416,436, OR = 0.981, 95% CI: 0.979 to 0.983).

Associations between neighbourhood deprivation and outcomes were also found in the subsample with information on dependence (n = 98,659), both when adjusting for dependence (Completion: OR = 0.966, 95% CI: 0.961 to 0.972; Success: OR = 0.985, 95% CI: 0.980 to 0.990) and when not adjusting for dependence (Completion: OR = 0.962, 95% CI: 0.957 to 0.968; Success: OR = 0.981, 95% CI: 0.976 to 0.986). Higher dependence was associated with reduced completion (OR = 0.915, 95% CI: 0.905 to 0.925) and success (OR = 0.917, 95% CI: 0.909 to 0.926).

Sensitivity analyses including only first supported attempts achieved results very similar for neighbourhood deprivation to those of the full sample (n = 364,397, Completion: OR = 0.951, 95% CI: 0.949 to 0.954; Success: OR = 0.967, 95% CI: 0.965 to 0.970).

## Discussion

Small but consistent associations between neighbourhood deprivation and reduced completion and reduced success of treatment outcomes were found while adjusting for confounding variables, including dependence. The association between success and neighbourhood deprivation remained even when looking only at those who had completed treatment. This indicates that reduced success was not fully explained by increased loss to follow-up and that lifting completion to a higher level for all clients would not be sufficient to raise success to the level of the least deprived clients. Indicators of individual deprivation and neighbourhood deprivation were each independently associated with outcomes. Using more recent data from a wider population than earlier reports, the present findings thus confirm reduced completion and success of treatment [22] in more deprived clients and concur with previous findings of independent effects of individual’s deprivation and neighbourhood deprivation [5–8].

Additional findings include that returning for repeated attempts with the services was far more common in more deprived groups, and although previous analyses found no association between this and quit success [26, 27], later attempts were associated with reduced success in the current analysis. This suggests that those who had been treated previously adhered to the treatment but nevertheless struggled to quit.

Regardless of neighbourhood deprivation, most clients used one of the two most effective medication options; however, with increased neighbourhood deprivation, use of varenicline declined in favour of combination NRT, which may be the least effective of the two in clinical practice [29], although not in clinical trials [13]. It is unclear why varenicline is used much less frequently with more deprived (and on average also more dependent) clients.

The analysis of the associations between intervention setting or type, included mainly as potential confounders, and the two different outcomes showed some interesting findings (Table 2). Compared with those supported in specialist clinics, clients supported in pharmacies were less likely to complete, but more likely to quit successfully which may indicate differential recording of clients in pharmacies. An opposing pattern was seen for clients supported in drop-ins or by unspecified intervention, who were more likely to have completed treatment but less likely to succeed than those seen in one-to-one appointments. This may underline the reduced effectiveness of less structured behavioural support even with good attendance.

A limitation of the data was that in some cases, more than one quit attempt was recorded for the same individual and this was more likely for more deprived clients. Importantly however, a large proportion of previous attempts will have taken place prior to the period of data collection and sensitivity analyses with only first supported attempts showed no discernible effect on the association between neighbourhood deprivation and outcomes. The large sample of services represents a strength of the study, particularly as the client and intervention characteristics were very similar to typical services available throughout England [30, 31].

Dependence is a strong predictor of quit success [32–35], and the present data indicated an association between neighbourhood deprivation and dependence that did not attenuate the association between neighbourhood deprivation and outcomes. Unfortunately, data on dependence were recorded inconsistently and for a minority of clients only, thus these findings have to be treated with some caution. Other possible barriers to successfully quitting smoking that were not assessed in the present data include higher smoking prevalence in the social context; a higher number of smoking friends for example is associated with less successful quitting [36, 37] and increased relapse [38].

Implications of these findings include that increased effort is needed to ensure that more disadvantaged smokers receive the most effective treatment, both in terms of behavioural support and medication. Combinations of support and medication as delivered by the stop smoking services have been shown to have a positive impact on inequalities when effectively targeted [39]. Even the most effective and well-targeted stop smoking support however needs to operate within a wider context of policies to reduce health inequality.

## Conclusions

Neighbourhood deprivation was associated with small but consistent reductions in completion and success of evidence-based interventions. These associations were not explained by intervention characteristics, demographics or dependence and reduced completion did not fully account for reduced success.

## Supporting Information

S1 Dataset (SAV)

## References

[1] Marmot M, Allen J, Bell R, Bloomer E, Goldblatt P, Consortium for the European Review of Social Determinants of H, et al WHO European review of social determinants of health and the health divide. Lancet. 2012;380(9846):1011–29. 10.1016/S0140-6736(12)61228-8 . 22964159

[2] Mackenbach JP, Kulhanova I, Menvielle G, Bopp M, Borrell C, Costa G, et al Trends in inequalities in premature mortality: a study of 3.2 million deaths in 13 European countries. Journal of epidemiology and community health. 2014 10.1136/jech-2014-204319 . 24964740

[3] Office for National Statistics. Do smoking rates vary between more and less advantaged areas? Available: http://www.ons.gov.uk/ons/rel/disability-and-health-measurement/do-smoking-rates-vary-between-more-and-less-advantaged-areas-/2012/sty-smoking-rates.html. 2014 [updated 12 Mar 2014, 2 Apr 2014].

[4] Marmot M, Bell R. Fair society, healthy lives. Public health. 2012;126 Suppl 1:S4–10. 10.1016/j.puhe.2012.05.014 . 22784581

[5] Kleinschmidt I, Hills M, Elliott P. Smoking behaviour can be predicted by neighbourhood deprivation measures. Journal of epidemiology and community health. 1995;49 Suppl 2:S72–7. 8594138 10.1136/jech.49.suppl_2.s72 PMC1060880

[6] Lakshman R, McConville A, How S, Flowers J, Wareham N, Cosford P. Association between area-level socioeconomic deprivation and a cluster of behavioural risk factors: cross-sectional, population-based study. J Public Health (Oxf). 2011;33(2):234–45. 10.1093/pubmed/fdq072 20884643 PMC3714999

[7] Shohaimi S, Luben R, Wareham N, Day N, Bingham S, Welch A, et al Residential area deprivation predicts smoking habit independently of individual educational level and occupational social class. A cross sectional study in the Norfolk cohort of the European Investigation into Cancer (EPIC-Norfolk). Journal of epidemiology and community health. 2003;57(4):270–6. 12646543 10.1136/jech.57.4.270 PMC1732421

[8] Turrell G, Hewitt BA, Miller SA. The influence of neighbourhood disadvantage on smoking cessation and its contribution to inequalities in smoking status. Drug Alcohol Rev. 2012;31(5):645–52. 10.1111/j.1465-3362.2012.00452.x . 22507105

[9] Schaap MM, Kunst AE, Leinsalu M, Regidor E, Ekholm O, Dzurova D, et al Effect of nationwide tobacco control policies on smoking cessation in high and low educated groups in 18 European countries. Tobacco control. 2008;17(4):248–55. 10.1136/tc.2007.024265 . 18483129

[10] Kotz D, West R. Explaining the social gradient in smoking cessation: it's not in the trying, but in the succeeding. Tobacco control. 2009;18(1):43–6. 10.1136/tc.2008.025981 18936053

[11] Partos TR, Borland R, Siahpush M. Socio-economic disadvantage at the area level poses few direct barriers to smoking cessation for Australian smokers: findings from the International Tobacco Control Australian cohort survey. Drug Alcohol Rev. 2012;31(5):653–63. 10.1111/j.1465-3362.2012.00427.x . 22385265 PMC4594828

[12] Reid JL, Hammond D, Boudreau C, Fong GT, Siahpush M, Collaboration ITC. Socioeconomic disparities in quit intentions, quit attempts, and smoking abstinence among smokers in four western countries: findings from the International Tobacco Control Four Country Survey. Nicotine Tob Res. 2010;12 Suppl:S20–33. 10.1093/ntr/ntq051 20889477 PMC2948137

[13] Cahill K, Stevens S, Perera R, Lancaster T. Pharmacological interventions for smoking cessation: an overview and network meta-analysis. Cochrane database of systematic reviews (Online). 2013; 5:CD009329 Epub 2013/06/04. 10.1002/14651858.CD009329.pub2 . 23728690 PMC8406789

[14] Stead LF, Lancaster T. Combined pharmacotherapy and behavioural interventions for smoking cessation. Cochrane database of systematic reviews (Online). 2012;10:CD008286 10.1002/14651858.CD008286.pub2 . 23076944

[15] Department of Health. Local Stop Smoking Services. London: Department of Health; 2012.

[16] West R, May S, West M, Croghan E, McEwen A. Performance of English stop smoking services in first 10 years: analysis of service monitoring data. BMJ (Clinical research ed). 2013;347:f4921 10.1136/bmj.f4921 . 23963106

[17] Hart JT. The inverse care law. Lancet. 1971;1(7696):405–12. . 4100731 10.1016/s0140-6736(71)92410-x

[18] Chesterman J, Judge K, Bauld L, Ferguson J. How effective are the English smoking treatment services in reaching disadvantaged smokers? Addiction (Abingdon, England). 2005;100 Suppl 2:36–45. Epub 2005/03/10. 10.1111/j.1360-0443.2005.01026.x . 15755261

[19] Bauld L, Judge K, Platt S. Assessing the impact of smoking cessation services on reducing health inequalities in England: observational study. Tobacco control. 2007;16(6):400–4. Epub 2007/12/01. 10.1136/tc.2007.021626 18048617 PMC2807194

[20] Brown T, Platt S, Amos A. Equity impact of European individual-level smoking cessation interventions to reduce smoking in adults: a systematic review. Eur J Public Health. 2014 10.1093/eurpub/cku065 . 24891458

[21] Bauld L, Chesterman J, Judge K, Pound E, Coleman T. Impact of UK National Health Service smoking cessation services: variations in outcomes in England. Tobacco control. 2003;12(3):296–301. Epub 2003/09/06. 10.1136/tc.12.3.296 12958391 PMC1747752

[22] Hiscock R, Judge K, Bauld L. Social inequalities in quitting smoking: what factors mediate the relationship between socioeconomic position and smoking cessation? J Public Health (Oxf). 2011;33(1):39–47. Epub 2010/12/24. 10.1093/pubmed/fdq097 . 21178184

[23] Lam TH, Abdullah AS, Chan SS, Hedley AJ, Hong Kong Council on Smoking and Health Smoking Cessation Health Centre (SCHC) Steering Group. Adherence to nicotine replacement therapy versus quitting smoking among Chinese smokers: a preliminary investigation. Psychopharmacology. 2005;177(4):400–8. 10.1007/s00213-004-1971-y . 15289997

[24] The English Indices of Deprivation 2010. 2011. Available: http://www.communities.gov.uk/publications/corporate/statistics/indices2010. Accessed 29 November 2011

[25] Hiscock R, Murray S, Brose LS, McEwen A, Bee JL, Dobbie F, et al Behavioural therapy for smoking cessation: The effectiveness of different intervention types for disadvantaged and affluent smokers. Addictive behaviors. 2013;38(11):2787–96. 10.1016/j.addbeh.2013.07.010 23954946 PMC3776925

[26] NHS choices. Help with health costs. Available: http://wwwnhsuk/nhsengland/Healthcosts/pages/Prescriptioncostsaspx. 2015; Accessed 21August 2015.

[27] West R, Hajek P, Stead L, Stapleton J. Outcome criteria in smoking cessation trials: proposal for a common standard. Addiction (Abingdon, England). 2005;100(3):299–303. 10.1111/j.1360-0443.2004.00995.x . 15733243

[28] Field A. Discovering Statistics Using SPSS. 2nd ed. London: Sage 2005.

[29] Brose LS, West R, Stapleton JA. Comparison of the effectiveness of varenicline and combination nicotine replacement therapy for smoking cessation in clinical practice. Mayo Clin Proc. 2013;88(3):226–33. Epub 2013/03/16. 10.1016/j.mayocp.2012.11.013 . 23489449

[30] The NHS Information Centre. Statistics on NHS Stop Smoking Services: England, April 2009 –March 2010. Leeds: The Health and Social Care Information Centre; 2010.

[31] The NHS Information Centre. Statistics on NHS Stop Smoking Services: England, April 2011 –March 2012. Leeds: The Health and Social Care Information Centre; 2012.

[32] Ferguson J, Bauld L, Chesterman J, Judge K. The English smoking treatment services: one-year outcomes. Addiction (Abingdon, England). 2005;100 Suppl 2:59–69. Epub 2005/03/10. 10.1111/j.1360-0443.2005.01028.x . 15755262

[33] Transdisciplinary Tobacco Use Research Center Tobacco D, Baker TB, Piper ME, McCarthy DE, Bolt DM, Smith SS, et al Time to first cigarette in the morning as an index of ability to quit smoking: implications for nicotine dependence. Nicotine Tob Res. 2007;9 Suppl 4:S555–70. 10.1080/14622200701673480 18067032 PMC2933747

[34] Chandola T, Head J, Bartley M. Socio-demographic predictors of quitting smoking: how important are household factors? Addiction (Abingdon, England). 2004;99(6):770–7. 10.1111/j.1360-0443.2004.00756.x . 15139875

[35] Vangeli E, Stapleton J, Smit ES, Borland R, West R. Predictors of attempts to stop smoking and their success in adult general population samples: a systematic review. Addiction (Abingdon, England). 2011;106(12):2110–21. Epub 2011/07/15. 10.1111/j.1360-0443.2011.03565.x . 21752135

[36] Hitchman SC, Fong GT, Zanna MP, Thrasher JF, Laux FL. The Relation Between Number of Smoking Friends, and Quit Intentions, Attempts, and Success: Findings From the International Tobacco Control (ITC) Four Country Survey. Psychology of addictive behaviors: journal of the Society of Psychologists in Addictive Behaviors. 2014 10.1037/a0036483 . 24841185 PMC4266625

[37] Zhou X, Nonnemaker J, Sherrill B, Gilsenan AW, Coste F, West R. Attempts to quit smoking and relapse: factors associated with success or failure from the ATTEMPT cohort study. Addictive behaviors. 2009;34(4):365–73. 10.1016/j.addbeh.2008.11.013 . 19097706

[38] Herd N, Borland R, Hyland A. Predictors of smoking relapse by duration of abstinence: findings from the International Tobacco Control (ITC) Four Country Survey. Addiction (Abingdon, England). 2009;104(12):2088–99. 10.1111/j.1360-0443.2009.02732.x . 19922574 PMC4517970

[39] Amos A, Bauld L, Hill S, Platt S, Robinson J. Tobacco control, inequalities in health and action at the local level in England. York: Public Health Research Consortium; 2011.

